# Natural language processing for the automated detection of intra-operative elements in lumbar spine surgery

**DOI:** 10.3389/fsurg.2023.1271775

**Published:** 2023-12-18

**Authors:** Sayan Biswas, Lareyna McMenemy, Ved Sarkar, Joshua MacArthur, Ella Snowdon, Callum Tetlow, K. Joshi George

**Affiliations:** ^1^ Faculty of Biology, Medicine and Health, University of Manchester, Manchester, United Kingdom; ^2^ College of Letters and Sciences, University of California, Berkeley, CA, United States; ^3^ Division of Data Science, The Northern Care Alliance NHS Group, Manchester, United Kingdom; ^4^ Department of Neurosurgery, Manchester Centre for Clinical Neurosciences, Salford Royal Hospital, Manchester, United Kingdom

**Keywords:** clips, dural tears, natural language processing, sutures, spine surgery, wound drains

## Abstract

**Background:**

The aim of this study was to develop natural language processing (NLP) algorithms to conduct automated identification of incidental durotomy, wound drains, and the use of sutures or skin clips for wound closure, in free text operative notes of patients following lumbar surgery.

**Methods:**

A single-centre retrospective case series analysis was conducted between January 2015 and June 2022, analysing operative notes of patients aged >18 years who underwent a primary lumbar discectomy and/or decompression at any lumbar level. Extreme gradient-boosting NLP algorithms were developed and assessed on five performance metrics: accuracy, area under receiver-operating curve (AUC), positive predictive value (PPV), specificity, and Brier score.

**Results:**

A total of 942 patients were used in the training set and 235 patients, in the testing set. The average age of the cohort was 53.900 ± 16.153 years, with a female predominance of 616 patients (52.3%). The models achieved an aggregate accuracy of >91%, a specificity of >91%, a PPV of >84%, an AUC of >0.933, and a Brier score loss of ≤0.082. The decision curve analysis also revealed that these NLP algorithms possessed great clinical net benefit at all possible threshold probabilities. Global and local model interpretation analyses further highlighted relevant clinically useful features (words) important in classifying the presence of each entity appropriately.

**Conclusions:**

These NLP algorithms can help monitor surgical performance and complications in an automated fashion by identifying and classifying the presence of various intra-operative elements in lumbar spine surgery.

## 1. Introduction

Administrative, billing, and coding tasks are a major source of financial and economic burden on healthcare systems worldwide (1). With the increase in healthcare and labour costs in recent years, major health systems are shifting towards minimising financial expenditure while maximising patient care. A key component in this process is optimising the clinical coding pipeline by reducing the burden on labour with limited manual review and intervention. The clinical coding process involves transforming medical records, usually presented as free text written by clinicians, into structured codes using the standardised Current Procedural Terminology (CPT) and the International Statistical Classification of Diseases (ICD) codes. The purpose of such clinical coding is to characterise the use of hospital services, document patient outcomes, and quantify clinical and surgical practices to allow for optimal financial reimbursement and to inform healthcare service planning and policy (2, 3).

Natural language processing (NLP) is a domain of machine learning that focuses on the analysis of structured and unstructured free text. NLP techniques are well suited for clinical coding due to their ability to analyse free text in real time with great precision. In the United Kingdom, the General Medical Council states that maintaining accurate and detailed clinical documentation is essential across all specialties for good medical practice (4), in addition to providing information for research, audits, and medicolegal records (5, 6). The current epidemic of defensive practice due to fear of medicolegal repercussions has had an extensive impact on neurosurgical documentation practices, resulting in more detailed documentation of procedures (7). Despite guidelines being available for the documentation of operative notes (8), many studies have demonstrated the inadequate quality of operative notes with much salient information missing, including the nature of the surgery, indication of surgery, estimated blood loss, incidence of complications, and postoperative instructions (6, 9–11). Such non-standardised documentation can lead to greater manual review times, making the extraction of relevant information more labour-intensive. The creation of accurate NLP algorithms trained on a large number of heterogeneous documents can be used to supplement the current clinical coding process, reducing the need for extensive and tedious manual reviews.

Spine surgery comprises the majority of operative cases in neurological surgery. Incidental durotomy, lumbar drains, and type of skin closure (sutures or clips) are important elements included in operative notes and are associated with patient outcomes, and therefore accurate documentation is vital to inform best clinical practice (12–16). At present, CPT and ICD-10 codes are used to identify incidental durotomies and “dural tears” within operative notes. However, these modalities have been shown to lack sensitivity, resulting in the underreporting of these complications (17–19). To the best of our knowledge, no such codes exist for the identification of the use of drains or wound closure technique used. Hence, the aim of this study is to develop NLP algorithms to conduct automated surveillance for identification of incidental durotomy, wound drains, and the use of sutures or skin clips for wound closure, in free text operative notes of patients following lumbar surgery. Towards this, in this study we attempted to evaluate if NLP techniques could be harnessed to analyse operative notes to detect the three important elements of spine surgery: incidental durotomy, the use of wound drains, and type of skin closure (suture or clips).

## 2. Materials and methods

### 2.1. Guidelines

The following guidelines were followed in this study: the *Journal of Medical Internet Research* (JMIR) Guidelines for Developing and Reporting Machine Learning Predictive Models in Biomedical Research, and the Transparent Reporting of Multivariable Prediction Models for Individual Prognosis or Diagnosis (TRIPOD) checklist (20, 21).

### 2.2. Data source and outcome measure

A single tertiary neurosurgical centre retrospective case series analysis was conducted for all patients who underwent lumbar spine surgery between January 2015 and June 2022. The inclusion criteria for this study were as follows: (1) patient age more than or equal to 18 years, (2) patient underwent a primary lumbar discectomy and/or decompression at any lumbar level, and (3) availability of index surgery operation notes in our electronic health records. The exclusion criteria included any patients with incomplete data and patients who underwent primary lumbar discectomy and/or fusion. The hospital's electronic patient records were examined and a total of 1,177 patients were identified. Each patient's operation note was then blinded and extracted in an anonymised manner. Our study was approved by the local hospital's institutional review board because of the retrospective and anonymised operative note data collection method. The study was registered as a health improvement project with the requirement for patient consent being waived. All methods were conducted in accordance with local and national guidelines and regulations.

Along with the operation notes, the age (continuous) and gender of the patient (male or female) were also collected as independent variables. There were three primary outcomes for each operation note: (1) the presence of intra-operative durotomies (binary outcome), (2) the placement of wound drains (binary outcome), and (3) the use of clips or sutures for skin closure (binary outcome). The terms durotomies and dural tears are used interchangeably in this paper. Each patient's operation note was reviewed and annotated by blinded researchers (LM and SB) who were not involved in the care of these patients. The results of each outcome category were then verified by the senior author.

### 2.3. Data pre-processing

**Figure 1:**
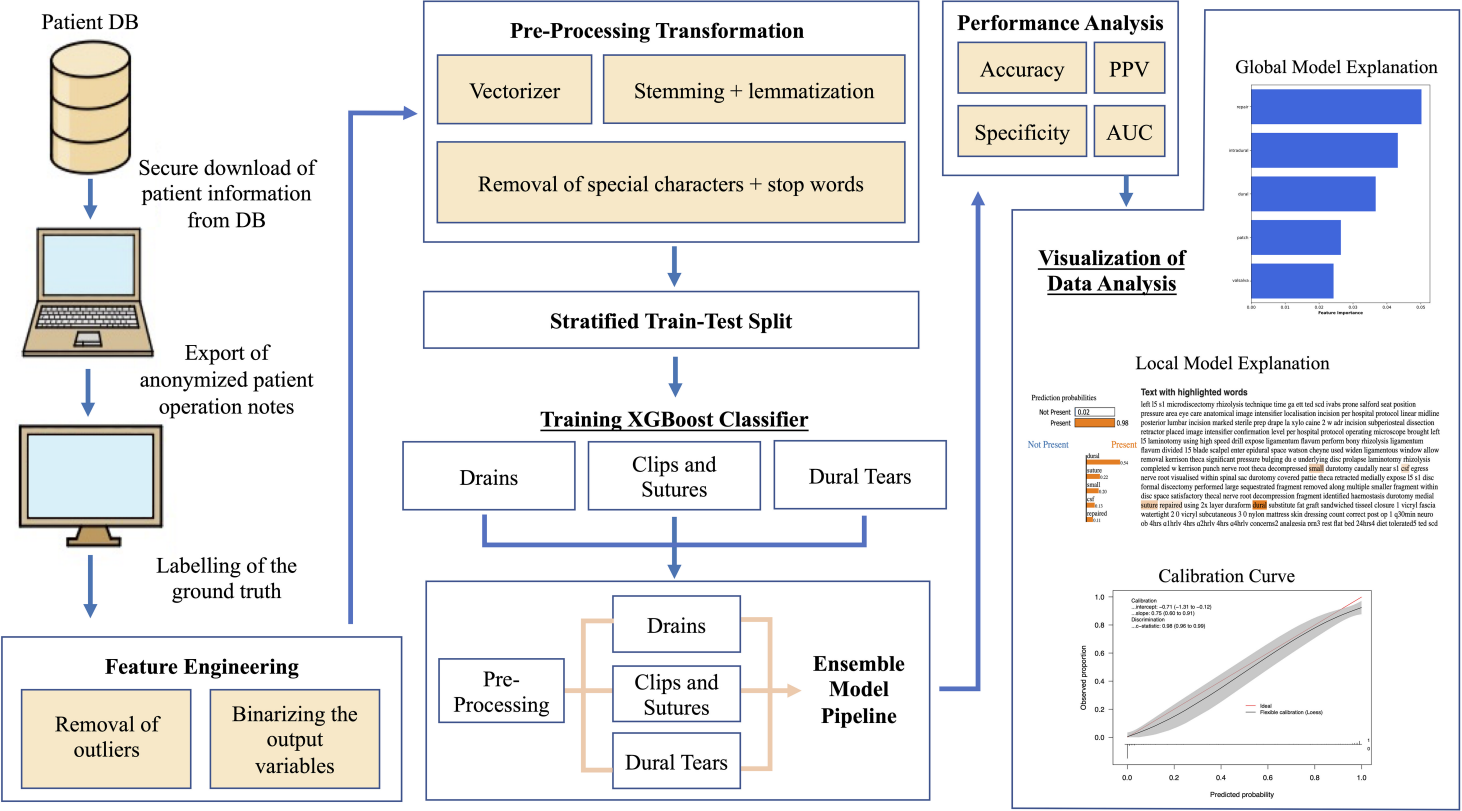
Data acquisition, processing, analysis, and visualisation pipeline. DB, database.

The data acquisition, pre-processing, model development, and evaluation pipeline have been highlighted in Figure 1. The dataset was initially cleaned with a custom data-cleaning function that consisted of the removal of special characters retrieved from the Natural Language Toolkit (NLTK) such as “@/{$#%&” and stopwords including “*and*”, “*or*”, and “the”. These words do not carry significant meaning or information in text analysis tasks, hence their removal helps to de-noise the text data resulting in the better efficiency and performance of NLP models. Stemming and lemmatisation are two common techniques used in the data-cleaning function, both of which aim to normalise words by reducing them to their base or root forms. Stemming achieves this by removing any suffixes at the end of a word, while lemmatisation is the process of reducing a word to its base or dictionary form (known as the lemma) while taking into account the context and part of speech of the word.

Lastly, the CountVectorizer library function was used to pre-process the cleaned data. By default, CountVectorizer uses the “term frequency” weighting for single tokenisation, which means it represents each word by the number of times it appears in a document. This results in a document-term matrix where each element represents the frequency of a particular word in a specific document. The resulting matrix is then used as the input to various machine learning algorithms such as clustering, classification, and topic modelling. By representing text data in a numerical format, the CountVectorizer enables machine learning (ML) algorithms to process and analyse textual data, which would otherwise be difficult due to the unstructured nature of natural language.

### 2.4. Model development

An 80:20 training–testing split was carried out on the total cohort of 1,177 patients, with 942 patients in the training set and 235 patients in the testing set. The datasets were stratified for the outcome variables to account for class imbalances. An extreme gradient-boosting (XGBoost) NLP classifier was developed to predict each outcome category. XGBoost was selected as the classifier of choice owing to a number of factors: (1) its ability to handle high-dimensional feature spaces such as word to vector embeddings used in NLP, (2) the ability to handle and adjust for sparse and imbalanced datasets using weighted loss functions and subsampling, (3) its faster computational run time and scalability, and (4) explicit feature importance calculation for each input attribute (11, 22). Three individual models were created for identifying each outcome category, and the outputs from the models were concatenated to produce a multilabel ensemble output with the predicted probabilities for each outcome. The three ML models will be referred to as the dural tear, drains, and clips vs. sutures models in this paper. An iterative process termed Grid Search was used to optimise the model hyperparameters. In grid search, a predefined set of hyperparameter values is defined, and the model is trained and evaluated on all possible combinations of these values to achieve the highest level of accuracy.

The models were trained on fivefold stratified K-fold cross-validation with five repeats on the training dataset. The training and testing datasets were stratified by each of the outcome categories to standardise the class imbalances within our outcome variables and provide us with the best overall performance results for the models. The performance of the models were evaluated via five performance metrics on the training and testing sets: accuracy, precision/positive predictive value (PPV), specificity, area under the receiver-operating curve (AUC)/discrimination, and the Brier score loss. All metrics were bootstrapped with 1,000 resamples to derive the associated 95% confidence intervals (CIs). Each model was then calibrated on the testing set. Calibration refers to how well a model's predicted probabilities align with the true observed probabilities in the study population. This is evaluated using a calibration curve, which is ideally a 45° straight line starting from the origin, with a slope of 1 (indicating the spread of the model's estimated probabilities over the observed probabilities), and an intercept of 0 (indicating how much the model tends to over- or underestimate the true probability). In this study, the preferred method of calibration was Platt scaling or sigmoid binned calibration, which involved dividing the probability range into 10 bins and evaluating the shape of the calibration curve, as well as its slope, intercept, and the Brier score loss metric. In addition, the decision curve analysis (DCA) was used to evaluate and plot the clinical benefit of using the NLP algorithms to predict the presence of each outcome variable over a wide range of predicted threshold probabilities. The DCA illustrates the net benefit defined as the number of true positives detected for each outcome class when using the NLP algorithms on individual patient operation notes.

A model-specific global feature importance analysis was conducted on the trained models via retrieval of each model's relative feature weights that were averaged across all training folds. Furthermore, the Local Interpretable Model-agnostic Explanation analysis was performed to predict and highlight the important features on an individual patient operation note level.

### 2.5. Statistical analysis

All statistical analyses were conducted using IBM SPSS software (Statistical Package for the Social Science; SPSS Inc., Chicago, IL, USA) Version 25 for Mac, Microsoft Excel (Office 365, Microsoft, Seattle, WA, USA), and the R coding language (R Foundation for Statistical Computing, Vienna, Austria). Histogram plots and the Kolmogorov–Smirnov test were utilised for tests of normality for the continuous variables. The chi-squared tests were used to compare all categorical variables, and the independent samples *t*-test was used to compare the means of the continuous variables. Temporal trend analysis with a linear line of best fit was conducted for all variables, within our retrospective observation time period. A *p*-value <0.05 was considered statistically significant.

## 3. Results

### 3.1. Cohort demographics

A total of 1,177 patients were included in the study, with 942 patients in the training set and 235 patients in the testing set. Table 1 demonstrates the total cohort demographics. The average age of the cohort was 53.900 ± 16.153 years, with a female predominance of 616 patients (52.3%). The rates of intra-operative durotomy and the use of wounds drains were 9.9% (117/1,177) and 31.6% (373/1,177), respectively. Overall, the use of sutures [710 (60.3%)] was more common for skin closure compared with the use of metal surgical clips [458 (38.9%)]. The inter-variable comparative analysis (Table 2) demonstrated a significant relationship between increasing patient age and the use of sutures (*p-*value = 0.001). We also noted that with an ageing population, the operative age of our patients significantly increased over our observation period (*p-*value = 0.013). There was also a statistically significant relationship between the use of sutures for skin closure in cases with intra-operative dural tears and wound drains (*p-*value < 0.001). However, there was no statistically significant relationship between the use of wound drains and the presence of intra-operative dural tears (*p-*value = 0.554).

**Table 1:** Cohort demographics of the total patient cohort.

	Total cohort (*n* = 1,177)
Age	53.900 ± 16.153
Sex	
Female	616 (52.3%)
Male	561 (47.7%)
Drain(s)	
Yes	373 (31.6%)
No	801 (67.8%)
Closure	
Clips	458 (38.9%)
Sutures	710 (60.3%)
Dural tear(s)	
Yes	117 (9.9%)
No	1,060 (90.1%)

**Table 2:** Inter-variable statistical correlation analysis using *t*-tests for continuous variables and Chi-square tests for categorical variables.

	*p*-value					
	Age	Sex	Drains	Closure	Dural tear(s)	Year of surgery
Age		0.137	0.283	0.001^a^	0.501	0.013^a^
Sex						
Female	0.137		0.278	0.294	0.217	0.322
Male						
Drain(s)						
Yes	0.283	0.278		<0.001^a^	0.554	0.906
No						
Closure						
Clips	0.001^a^	0.294	<0.001^a^		<0.001^a^	0.017^a^
Sutures						
Dural tear(s)						
Yes	0.501	0.217	0.554	<0.001^a^		0.853
No						

^a^ Statistically significant *p*-value.

### 3.2. Temporal trend analysis

The Mann–Kendall test was used to analyse the temporal trends of the variables across our observation time period as shown in Supplementary Figure S1. During the study period, the total number of lumbar discectomies and/or decompressions decreased significantly from 220 surgeries in 2015 to 56 in the first half of 2022 (−112 estimated in a year) (tau = −0.929, *p-*value = 0.002). This decline was observed in all the years with the exception of 2019, which saw an increase of one operation from the previous year. It was noted that there was also a decrease in all spinal procedures post COVID-19, which may account for the decrease. The frequency of intra-operative durotomies/dural tears did decrease over the study period; however, no statistical significance was observed (tau = −0.286, *p-*value = 0.386), with rates ranging from 5.8% to 14.2%. The frequency of intra-operative placement of wound drains also statistically significantly increased over the study period, rising from 18.6% in 2015 to 41.1% in 2022 (tau = 0.643, *p-*value = 0.035). The preferred method of skin closure also changed over the study period, demonstrating a preference for closure with sutures in later years (tau = 0.5, *p-*value = 0.108) with a rise from 54% in 2015 to 75% in 2022. We observed an exact yet complementary decrease in the use of surgical clips for skin closure over the years (tau = −0.5, *p-*value = 0.108).

### 3.3. Model performance

**Figure 2:**
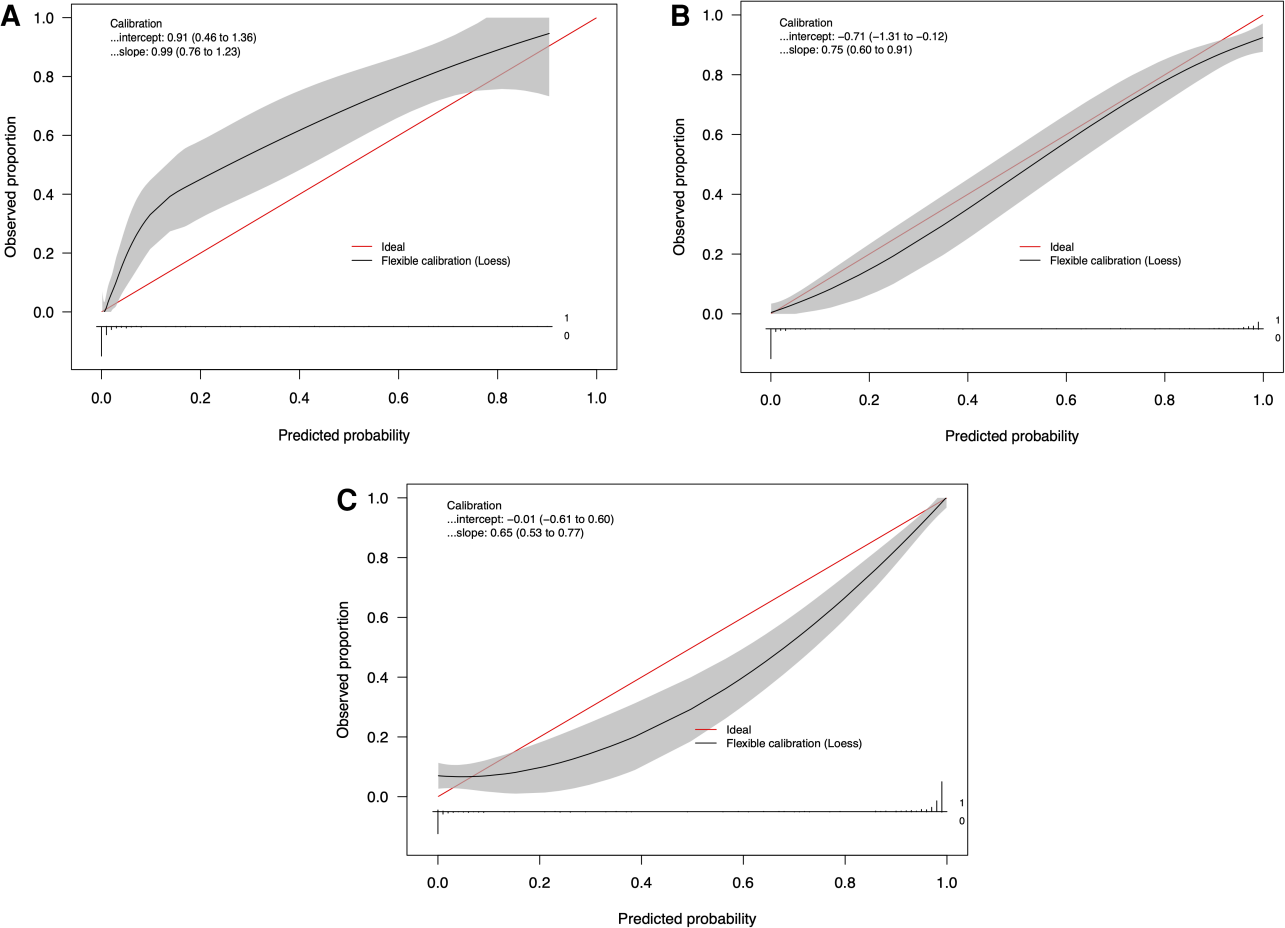
Calibration curves of the natural language processing models for identifying (**A**) dural tears, (**B**) wound drains, and (**C**) clips vs. sutures, in the testing set (*n* = 235).

**Figure 3:**
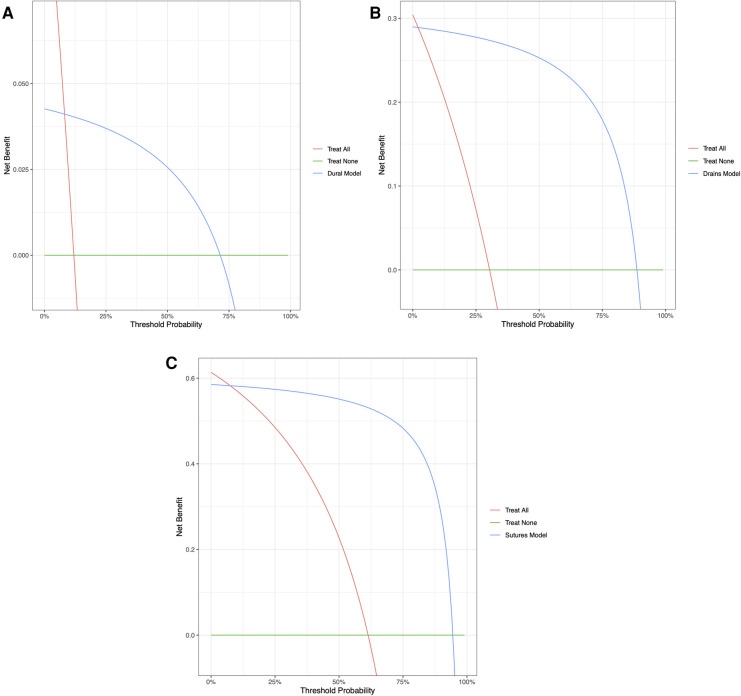
Decision curve analyses comparing expected clinical net benefit of the three models: (**A**) dural tears, (**B**) wound drains, and (**C**) clips vs. sutures, on the testing set.

Table 3 provides the performance metrics for the three ML models on the testing dataset. The dural tears model achieved an accuracy of 91.7615 (95% CI: 88.636–94.602), a PPV of 84.211% (95% CI: 80.667–90.000), a specificity of 99.032% (95% CI: 96.959–99.750), and an AUC of 0.946 (95% CI: 0.917–0.970). The drains model achieved an accuracy of 94.894% (95% CI: 92.330–97.160), a PPV of 88.696% (95% CI: 82.308–94.000), a specificity of 94.694% (95% CI: 90.886–97.025), and an AUC of 0.950 (95% CI: 0.923–0.973). The clips vs. sutures model achieved an accuracy of 93.750% (95% CI: 91.193–96.307), a PPV of 94.495% (95% CI: 91.379–97.260), a specificity of 91.177% (95% CI: 84.770–95.153), and an AUC of 0.933 (95% CI: 0.923–0.973). Figure 2 shows the calibration curves for each of the models. The dural tears model had a propensity to underpredict the presence of a dural tear, with a Brier score loss of 0.082 (95% CI: 0.054–0.114), an intercept of 0.91 (95% CI: 0.46–1.36), and a slope of 0.99 (95% CI: 0.76–1.23). The drains model demonstrated excellent calibration across all predicted probabilities with a Brier score loss of 0.051 (95% CI: 0.028–0.076), an intercept of −0.71 (95% CI: −1.31 to −0.12), and a slope of 0.75 (95% CI: 0.60–0.91). The clips vs. sutures model demonstrated a tendency to overpredict the use of sutures for skin closure, with a Brier score loss of 0.063 (95% CI: 0.037–0.088), an intercept of −0.01 (95% CI: −0.61–0.60), and a slope of 0.65 (95% CI: 0.53–0.77). Lastly, the decision curve analysis on the testing set revealed that all NLP algorithms ensured greater clinical net benefit at all possible threshold probabilities relative to the default decisions of changes made for all or none patients (Figure 3).

**Table 3:** Performance metrics of the machine learning model on the testing set with 95% confidence intervals.

Model	Accuracy (%)	Precision/PPV (%)	Specificity (%)	AUC	Brier score loss
Testing set (*n* = 235)					
Dural tears	91.761 (88.636–94.602)	84.211 (80.667–90.000)	99.032 (96.959–99.750)	0.946 (0.917–0.970)	0.082 (0.054–0.114)
Drains	94.894 (92.330–97.160)	88.696 (82.308–94.000)	94.694 (90.886–97.025)	0.950 (0.923–0.973)	0.051 (0.028–0.076)
Clips vs. sutures	93.750 (91.193–96.307)	94.495 (91.379–97.260)	91.177 (84.770–95.153)	0.933 (0.923–0.973)	0.063 (0.037–0.088)

### 3.4. Model explainability

**Figure 4:**
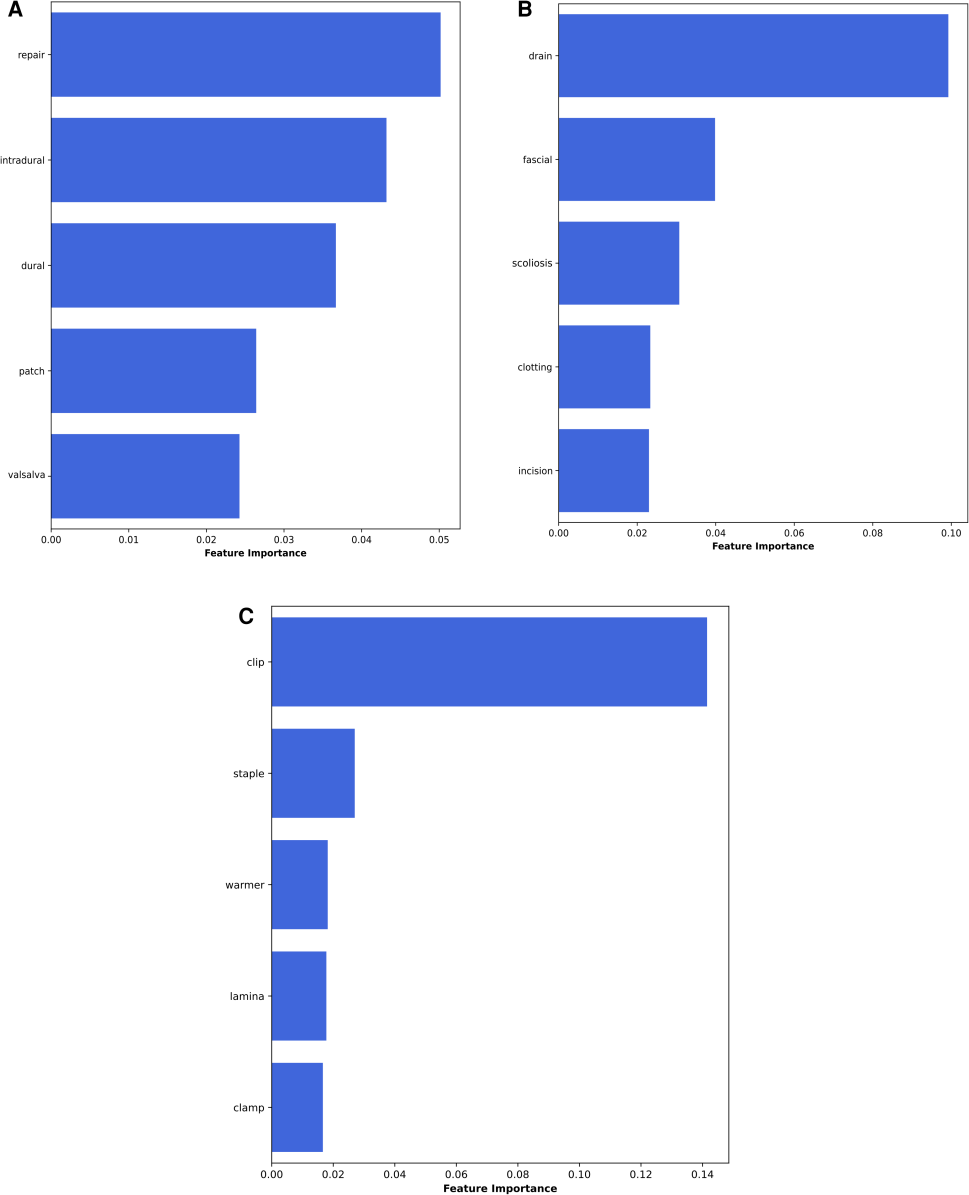
Global feature importance values for the NLP algorithms: (**A**) dural tears, (**B**) wound drains, and (**C**) clips vs. sutures.

**Figure 5:**
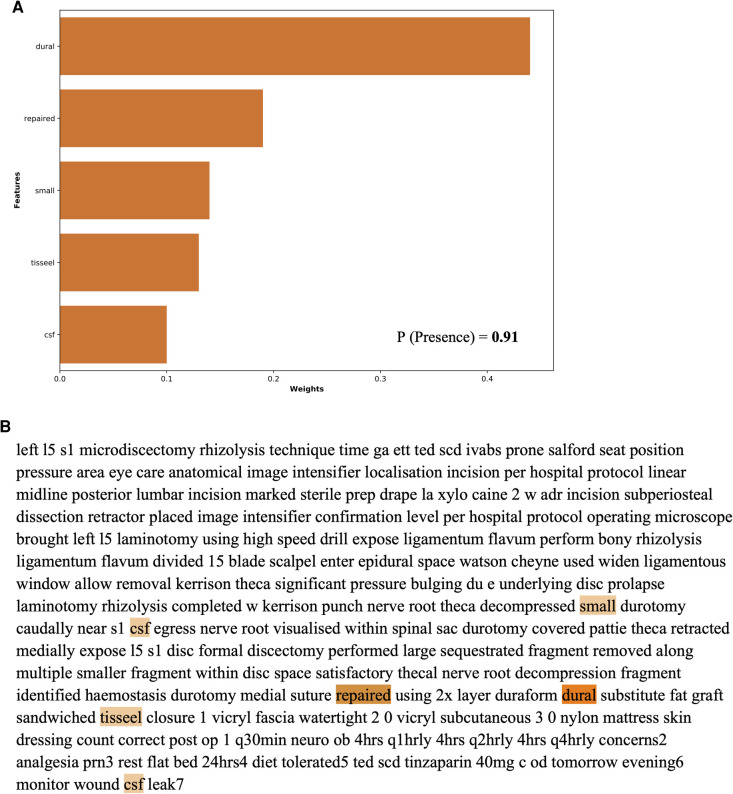
Local feature importance analysis for detecting dural tears in an example individual patient operation note as generated by the NLP algorithm.

The global feature importance calculations for the NLP algorithms are presented in Figure 4. These explanations highlight that for identification of an intra-operative durotomy, the five most meaningful features (words) are: “repair,” “intradural,” “dural,” “patch,” and “Valsalva.” The five most important features (words) for detecting the intra-operative placement of a lumbar drain are: “drain,” “fascial,” “scoliosis,” “clotting,” and “incision.” Similarly, for detecting whether surgical clips or traditional sutures were utilised for skin closure, the following five words were the most important: “clip,” “staple,” “warmer,” “lamina,” and “clamp.” In addition, the local feature importance analysis for an example patient level operation note demonstrates that the dural tear model is able to identify the five most important clinically meaningful features to detect the presence of an intra-operative dural tear (Figure 5). Interestingly, the local feature importance analysis for the drains and clips vs. sutures model demonstrated that the algorithm primarily searched only for the words “drain” and “clip,” respectively, to make the prediction, with the other aforementioned features possessing very little impact on the outcome.

## 4. Discussion

This study analysed the trends in the use of various intra-operative elements in spine surgery and developed NLP algorithms capable of reliably identifying these elements in operative notes. The automated identification of these elements can facilitate more efficient clinical coding and billing processes, help optimise hospital quality improvement and safety efforts, assist clinicians in auditing surgical practices, and guide overall resource allocation. This study demonstrates that our NLP algorithms are capable of reliably and accurately identifying the placement of intra-operative wound drains, the presence of incidental dural tears, and whether surgical clips or sutures were utilised for skin closure. This is the first ever study from a country with a public healthcare system that has demonstrated the feasibility of using automated NLP systems in operative notes to potentially guide both surgical practices and resource allocation.

The use of NLP techniques in spine surgery has seen a rise in the recent years and is projected to rapidly grow in the future (23, 24). The ability of NLP to perform precise automated surveillance of operative notes, to answer clinically relevant questions, serves to reduce the burden of time-intensive and error-prone reviews by clinical coders (25, 26). The delays in clinical coding within the National Health Service (NHS) impose a significant burden, with the potential for funding to be blocked if coding is not completed within a prerequisite timeframe (27). The average accuracy of this coding has been reported at approximately 83%, with large inter-study variability (28). Such problems exist in majority of healthcare systems worldwide and necessitate the development of automated techniques capable of facilitating these burdensome manual record review processes. Within this realm, Zaidat et al. have already developed an XLNet model capable of automatically generating CPT billing codes from operative notes for three specific surgical procedures: anterior cervical discectomy and fusion (ACDF), posterior cervical discectomy and fusion (PCDF), and cervical disc arthroplasty (CDA) (2). Such models have the potential to greatly reduce manual review/input, minimise errors in the coding process, and promote standardisation. Most recently, Shost et al. have also demonstrated a model capable of reliably identifying the type of spinal surgery performed via analysis of patient consent forms (29). The ability to rapidly classify surgical practices can be beneficial to both hospitals and the practicing surgeons. This will help track surgical volume, surgery-specific patient outcomes, and also provide trainees with a method of tracking individual surgical experience. In addition, NLP algorithms have also demonstrated predictive value in classifying lumbar spine imaging findings and in determining the need for surgical intervention in patients with low back pain via analysis of radiological and clinical reports (30). These examples highlight the importance of NLP techniques in improving the provision of patient care and demonstrate the clinical utility of such models in enhancing hospital and surgical practices.

Our NLP algorithms were developed to identify the presence of three important intra-operative factors that play a role in guiding the resource allocation and surgical practices of a neurosurgical department. In this study, the prevalence of incidental durotomy was 9.9%, in line with the recent literature on lumbar surgery (18, 31, 32). Our model demonstrated adequate discrimination and performance in identifying intra-operative dural tears and highlighted the use of clinically relevant features (words) to make its predictions. Previous studies by Karhade et al. have also been successful in the identification of incidental durotomy with an accuracy of 99%, surpassing the performance of CPT and ICD-10 codes, which demonstrated an accuracy of only 64% (18). Interestingly, however, the feature importance in their NLP algorithm showed different features compared with ours, further underscoring the potential variability in NLP algorithm performance across different cohorts that are geographically separated, and highlighting the need for broader validation studies (17). The importance of reliably identifying cases of intra-operative incidental durotomy is highlighted by evidence suggesting that patients with durotomies tend to have increased operative durations and inpatient length of stay (LOS) (33). Thus, accurate depiction of the rates of incidental durotomy can aid postoperative patient counselling, quantify surgical complication rates, and help track surgical performance.

For the wound drains model, our study demonstrated an accuracy of almost 95%. Previous studies have concluded that postoperative drains are currently being overused in spinal surgery, potentially imposing an increased risk of unnecessary complications, while not lending substantial benefit (34). Most notably, reports have suggested an elevated risk of surgical site infections (SSI) (35, 36), although this has been refuted by other papers (15, 37–39). Ho et al. interestingly report that both the absence of a wound drain and increased drainage when drains are used indicate an increased risk of delayed infection after posterior spine surgery (36). Walid et al. additionally found that the use of postoperative drains was linked to increased post-haemorrhagic anaemia, and a subsequent requirement of allogenic blood transfusions (40), which may impose greater costs to the healthcare system. Adogwa et al. have also demonstrated that patients with postoperative drains have a significantly longer LOS compared with patients with no drains (37). The combination of such factors highlights the importance of tracking and quantifying the use of drains in spine surgery, and therefore the development of our NLP algorithm will allow for its automated and reliable detection. The future application of this algorithm in tracking wound drain use and the associated SSI rates remains to be investigated.

Further, our clips vs. suture model demonstrated an accuracy of >93% accuracy, and our temporal trend analysis showed a preference for using sutures for wound closure. Various studies have concluded that suturing is more efficient when compared with the use of clips for good wound closure, resulting in lower rates of separation, prevention of SSI, and ultimately shorter hospital LOS (41, 42). Contrastingly, postoperative analysis of visual analogue pain scores comparing the use of clips to non-absorbable sutures have also demonstrated a significantly quicker and pain-free experience for patients with stapled wounds (43). From an economic perspective as well, studies have demonstrated that staples/clips are less expensive than sutures and that the financial gain appears to increase as laceration length increases (44). However, conflicting literature exists on the impact of sutures and clips on patients postoperatively (22), with the need for future robust randomised control trials to further investigate their effects. Nevertheless, such single-use surgical items are the largest contributors to the surgical carbon footprint and hence precise quantification of such use can guide both financial and environmental practices (45). Therefore, such automated NLP techniques can facilitate accurate data collection and analysis of the use of clips and sutures in neurosurgery. Nevertheless, the utility of this NLP algorithm in identifying and predicting postoperative LOS, risk of SSI, and the estimated carbon footprint after a surgery remains to be explored in a future study.

### 4.1. Limitations

Despite these results, the study has several limitations. First, this was a retrospective analysis at a single centre and therefore the development and testing of the NLP algorithms was geographically limited to a specific region. This raises questions about the algorithms’ generalisability and their performance in diverse linguistic and clinical contexts. Furthermore, the surgeons affiliated with the healthcare entities in the study likely share practices that influence the specific terminology used to document the various intra-operative characteristics, which could bias the results. Hence, future prospective and external validation of the algorithms needs to be performed to validate the clinical utility of the algorithms. In addition, there are other approaches that can be utilised to adapt our general model to geographically distinct regions. Geographically customisable models can be implemented via techniques such as federated learning and transfer learning. Federated learning enables the collaborative training of models across multiple centres without data sharing, preserving both privacy and centre-specific relationships and trends in the data. Transfer learning further facilitates rapid fine-tuning, which can efficiently adapt a base model to new regions by learning from small local datasets, boosting model performance and reliability. Secondly, though these models are able to reliably identify the outcomes of interest, a further manual review by clinical coders will still be required to exclude any cases of false positives or false negatives. Thus, the need for manual review will still exist, though with a considerably lower level of burden. Hence, multicentre, linguistically different validation studies in hospitals with varying coding/billing practices are required to determine the reliability of these models. Lastly, with the advent of state-of-the-art large language models such as Bidirectional Encoder Representations from Transformers and Generative Pre-trained Transformer models, the need for manual annotation of unstructured, free text data may exponentially reduce. These models are capable of independently performing named entity recognition and can understand the contextual nuances of each outcome of interest. For example, these models would be able to interpret the reason/context for using a drain, or the reason for a durotomy. Thus, in the future the goal would be to develop such models capable of functioning independently without the need for any manual annotation or review.

## 5. Conclusion

In conclusion, this study evaluated the feasibility and reliability of NLP algorithms in determining the presence of three intra-operative elements in lumbar spine surgery. We demonstrate that these NLP models possess great discriminative ability and accuracy in predicting the presence of wound drains, incidental dural tears, and the use of clips or sutures for wound closure. These models can help automate the clinical coding process, help optimise hospital quality improvement, and monitor surgical performance and practices. This is the first ever study from a country with a primarily public healthcare system that has demonstrated the feasibility of using automated NLP systems in operative notes to potentially guide both surgical practices and resource allocation.

## Data Availability

The original contributions presented in this study are included in the article/supplementary materials and further inquires should be directed to the corresponding author.

## Supplementary material

The Supplementary Material for this paper can be found online at: https://www.frontiersin.org/articles/10.3389/fsurg.2023.1271775/full#supplementary-material
