# Supplementary material for: Natural language processing for the automated detection of intra-operative elements in lumbar spine surgery
Source: Front Surg. 2023 Dec 18;10:1271775. doi: 10.3389/fsurg.2023.1271775 (PMC10757971; doi:10.3389/fsurg.2023.1271775)

Supplementary Figure 1: Temporal trend analysis of the change in the: **A)** number of spine surgeries, **B)** dural tears, **C)** clips, **D)** sutures and **E)** wound drains over the study time period.

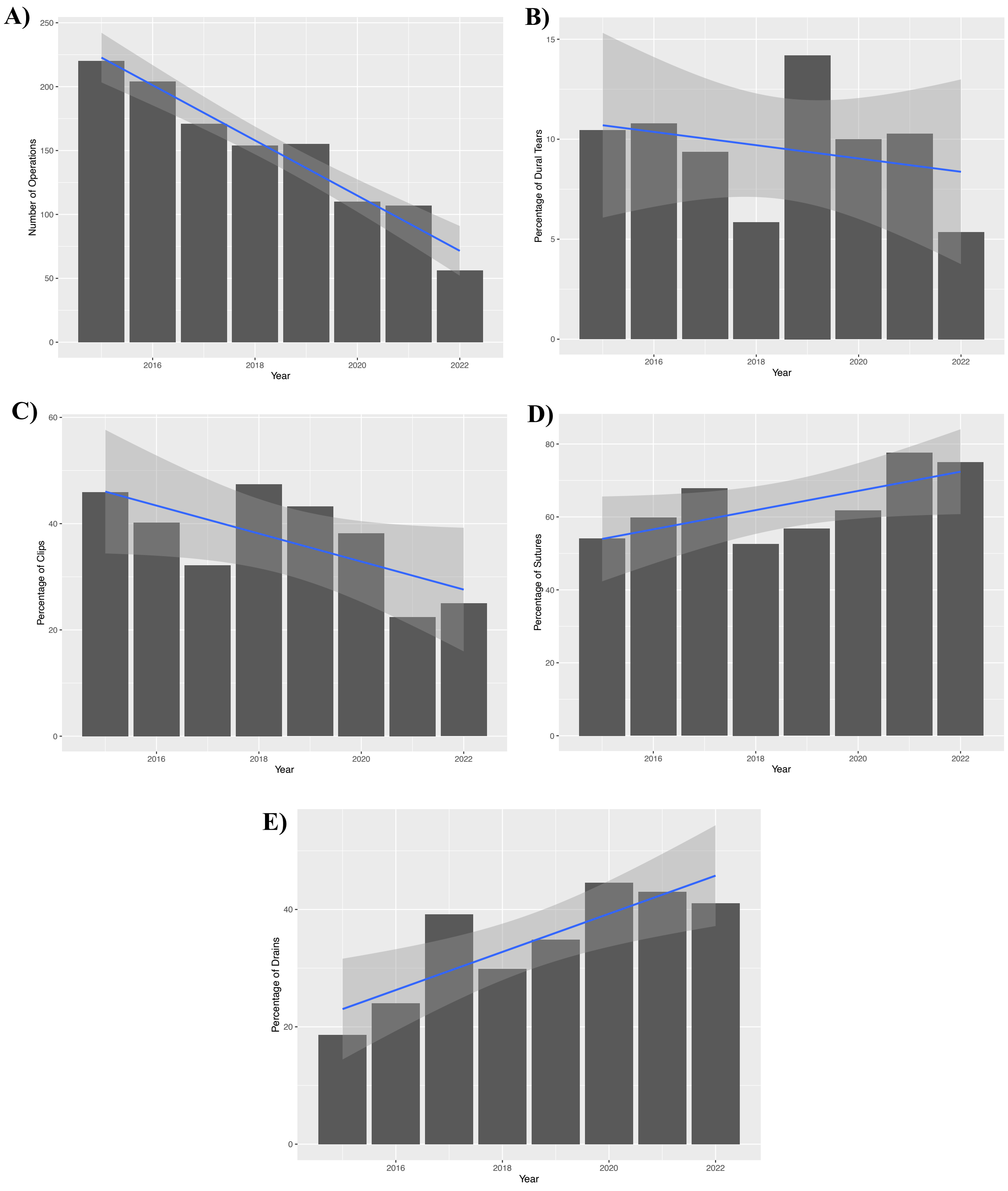

Supplement: Supplementary file 1 [file Image1.pdf]
